# 1329. Clinical Profile and Outcomes of Adults with Meningitis: A Prospective Observational Study from India

**DOI:** 10.1093/ofid/ofad500.1167

**Published:** 2023-11-27

**Authors:** Pankaj Sukhadiya, Maya Gopalakrishnan, Gopal Krishana Bohra, Kamlakant Shukla, M K Garg

**Affiliations:** All India Institute of Medical Sciences, Jodhpur, Rajasthan, India; All India Institute of Medical Sciences, Jodhpur, Rajasthan, India; AIIMS Jodhpur, Jodhpur, Rajasthan, India; All India Institute of Medical Sciences, Jodhpur, Rajasthan, India; AIIMS, Jodhpur, Rajasthan, India

## Abstract

**Background:**

Meningitis is a highly fatal disease, causing 33,337 deaths/year in India, > 50% of survivors develop sequelae -hearing loss, seizures, and cognitive impairment. Prior studies have been limited to pediatric populations or specific etiology. We explored the clinical profile, outcomes, and mortality risk factors in adult meningitis.

**Table 1:**
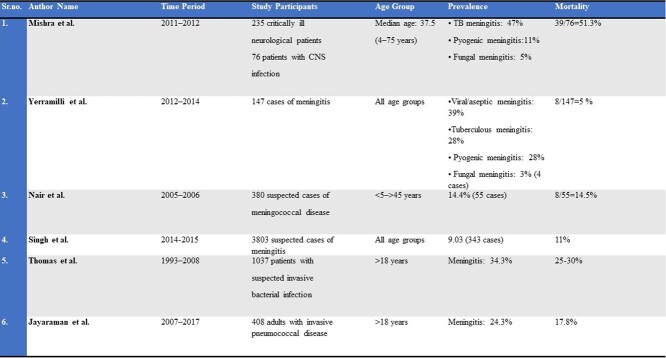
Previous studies on the prevalence and outcomes of meningitis in India

**Methods:**

Clinically suspected meningitis defined as 2 of 3 features- fever, headache, and focal/global central nervous system (CNS) dysfunction presenting to our centre were included from Mar 21-Sept 22, after consent and ethics approval. Patients were classified as acute/subacute based on the duration of illness of ≤ or > 5 days. Clinical details, Glasgow coma scale (GCS), cerebrospinal fluid parameters, and brain imaging were recorded. Modified Rankin Scale score (mRSS) was calculated at admission, discharge, and at 1 month. Multivariable regression analysis was done to identify mortality and morbidity risk factors.

**Results:**

Of 209 patients enrolled, 154 were diagnosed with meningitis, (57% acute 43% subacute) with viral (40%), tubercular (TBM) (36%), bacterial (12%), and others (12%). Mean age was 48.2 ± 20.5y, male: female ratio 1.6, 24% had the classical triad of fever, headache, and neck stiffness. Abnormal brain imaging was reported in 77%- MRI (97.5%) and CT (60%). Mortality was 29% (n=44) and 30% within TBM. Medical Research Council grade was distributed as grade 3(48%), grade 2 (32%) and grade 1(20%) in TBM. At admission, 44% had mRSS 4 (highest). The highest score at discharge and 1 month were 1 (25%) and 0 (32%) respectively. 105 patients developed complications, 70% CNS, 4% only extra CNS and 26% both; most common being drug induced hepatitis in TBM (n=29). Multivariable regression showed GCS ≤ 10, focal neurological deficits, sepsis at admission, thrombocytopenia (≤ 1,00,000/uL) and HsCRP ≥ 45 mg/dL, were significant mortality predictors, whereas low GCS at presentation predicted morbidity.

**Figure 1:**
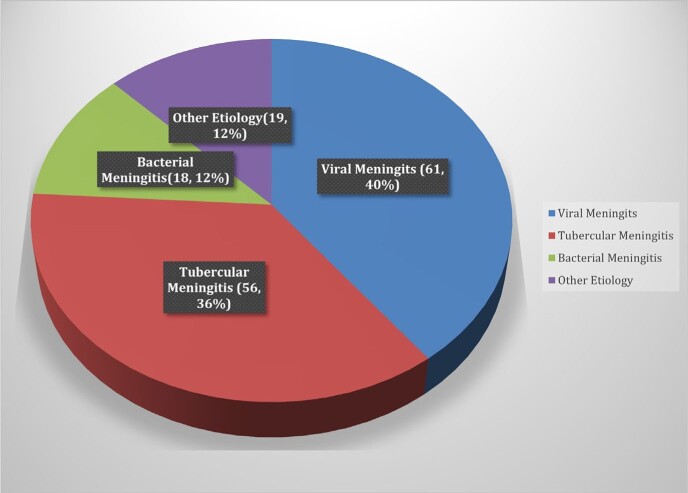
Frequency of etiology in meningitis patients (n=154)

**Figure 2:**
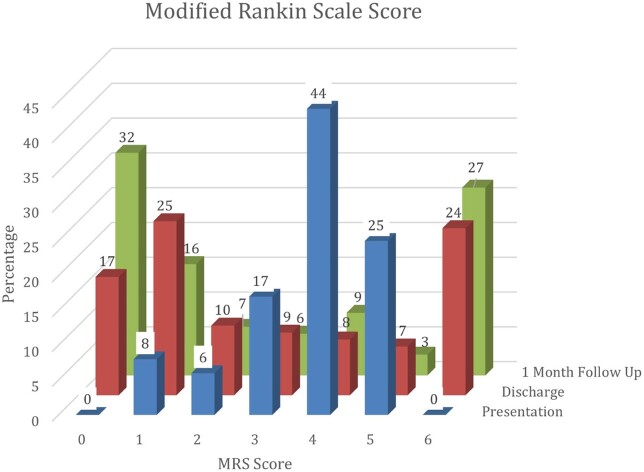
MRS Score at admission, on discharge and at 1 month follow up in meningitis patients (n=154)

**Table 2:**
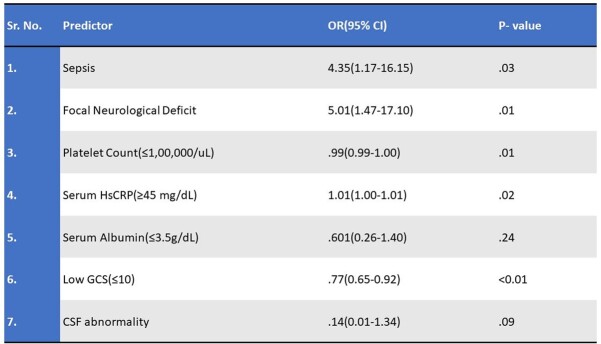
Multivariable analysis: Mortality predictors in meningitis patients (n=154). OR - Odds Ratio CI - Confidence Interval

**Conclusion:**

This is the first Indian study to explore the clinical profile and outcomes in adult meningitis. Viral meningitis was most frequent, while TBM had most fatalities. Our study shows that meningitis mortality is high even at tertiary care facilities in India. This highlights the need for reliable point of care diagnostics and better therapeutics.

**Table 3:**
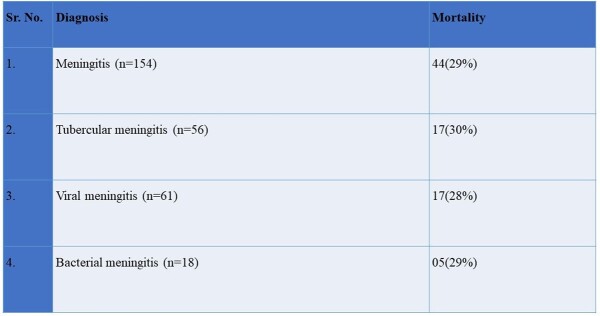
Mortality in enrolled patients with meningitis (n=154)

**Table 4:**
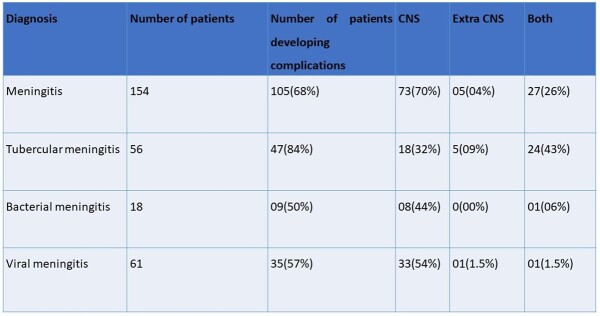
Frequency of complications within different meningitis etiologies (n=154)

**Disclosures:**

**All Authors**: No reported disclosures

